# Dynamics of sustained use and abandonment of clean cooking systems: study protocol for community-based system dynamics modeling

**DOI:** 10.1186/s12939-016-0356-2

**Published:** 2016-04-26

**Authors:** Praveen Kumar, Nishesh Chalise, Gautam N. Yadama

**Affiliations:** Brown School, Washington University in St. Louis, Campus Box 1196, One Brookings Drive, St. Louis, MO 63130 USA; Department of Social Work, Augsburg College, 2211 Riverside Avenue, Minneapolis, MN 55454 USA; Brown School, Washington University in St. Louis, Campus Box 1196, One Brookings Drive, St. Louis, MO 63130 USA

**Keywords:** Household air pollution, Sustained adoption, Community based system dynamics, Group model building, Clean cooking technologies, Dissemination & implementation

## Abstract

**Background:**

More than 3 billion of the world’s population are affected by household air pollution from relying on unprocessed solid fuels for heating and cooking. Household air pollution is harmful to human health, climate, and environment. Sustained uptake and use of cleaner cooking technologies and fuels are proposed as solutions to this problem. In this paper, we present our study protocol aimed at understanding multiple interacting feedback mechanisms involved in the dynamic behavior between social, ecological, and technological systems driving sustained use or abandonment of cleaner cooking technologies among the rural poor in India.

**Methods/Design:**

This study uses a comparative case study design to understand the dynamics of sustained use or abandonment of cleaner cooking technologies and fuels in four rural communities of Rajasthan, India. The study adopts a community based system dynamics modeling approach. We describe our approach of using community based system dynamics with rural communities to delineate the feedback mechanisms involved in the uptake and sustainment of clean cooking technologies. We develop a reference mode with communities showing the trend over time of use or abandonment of cleaner cooking technologies and fuels in these communities. Subsequently, the study develops a system dynamics model with communities to understand the complex sub-systems driving the behavior in these communities as reflected in the reference mode. We use group model building techniques to facilitate participation of relevant stakeholders in the four communities and elicit a narrative describing the feedback mechanisms underlying sustained adoption or abandonment of cleaner cooking technologies.

**Discussion:**

In understanding the dynamics of feedback mechanisms in the uptake and exclusive use of cleaner cooking systems, we increase the likelihood of dissemination and implementation of efficacious interventions into everyday settings to improve the health and wellbeing of women and children most affected by household air pollution. The challenge is not confined to developing robust technical solutions to reduce household air pollution and exposure to improve respiratory health, and prevent associated diseases. The bigger challenge is to disseminate and implement cleaner cooking technologies and fuels in the context of various social, behavioral, and economic constraints faced by poor households and communities.

**Trial registration:**

The Institutional Review Board of Washington University in St. Louis has exempted community based system dynamics modeling from review.

## Background

### Global Burden of disease due to household air pollution

Approximately 41 % of the world’s population—mainly the rural poor—continue to rely on the use of unprocessed biomass and solid fuels such as wood, charcoal, agricultural residues, and animal dung for cooking and heating. The release of significant carbonaceous aerosol emissions and particulate matter from traditional stoves using these solid fuels is a primary source of household air pollution (HAP), which causes the premature deaths of over 4 million people across the world [1]. Exposure to HAP is particularly high for women and young children. Nearly 50 % deaths from acute lower respiratory infections in underdeveloped countries among children who are less than 5 years old are attributed to HAP [1]. Approximately 3.8 million premature deaths due to non-communicable diseases (NCDs) including chronic obstructive pulmonary disease (COPD), stroke, heart disease, and lung cancer are attributed to exposure to HAP [1]. Approximately 4.3 million premature deaths occurred globally in 2012 alone as a result of HAP. [2]. Low-and middle-income countries (LMICs) in South Asia and Africa are particularly at high risk due to HAP [2]. In India, for example, more than 145 million poor households are directly exposed to HAP, and HAP is believed to be responsible for approximately 900,000 deaths in India each year [3, 4]. Moreover, the drudgery and time spent collecting biomass deters the poor, and especially women, from other productive income-generating activities [5]. Women are also vulnerable to physical injuries from carrying heavy loads of biomass [6–8]. They also face enhanced risk of sexual harassment and assault, while collecting biomass from forests [9]. The deleterious effects of HAP on public health, the environment, and economic well-being—again, especially of women—is a complex problem and a significant challenge.

### Cleaner cooking systems: the challenges of sustained use and abandonment

Sustained adoption of cleaner cooking technologies and fuels (e.g., cleaner cook stoves, biogas digesters, and LPG) in place of traditional stoves are proposed as a solution to address the challenges associated with HAP [6, 8, 10–12]. Epidemiological studies indicate that these cleaner cooking systems generate substantial public health and environmental dividends [6, 8, 11, 13, 14]. However, dissemination and implementation of cleaner cooking systems (cleaner stoves and cleaner fuels) have presented their own set of persistent challenges. First, there has been low levels of uptake and sustained use of these technologies by poor communities across the globe [6, 11]. Second, even when households, and communities take up cleaner cooking systems, they are co-located or stacked alongside traditional cooking technologies, diminishing the health returns from uptake of cleaner cooking [15]. Stacking in this context is where households begin using cleaner cooking technologies and cleaner fuels but in combination with their traditional cooking methods and fuels, never completely replacing solid fuels and traditional cooking methods with cleaner cooking technologies and fuels [15]. Epidemiological studies show that a sustained and exclusive use of clean cooking systems with a complete abandonment of traditional cooking practices is pivotal for realizing the expected health benefits in these communities [8]. Thus, despite the promise of these cleaner cooking systems, health benefits are substantially compromised due to stacking of cleaner cooking ways with traditional cooking systems. High abandonment rate and stacking of cleaner cooking systems has been a central issue in the persistence of HAP and associated health problems [6, 8, 11, 15].

### Previous research on the adoption and sustained use of cleaner cooking systems: Key limitations

The empirical literature addressing the sustained adoption of cleaner cooking systems remain narrow, scattered, and unstructured. A review of available research in these areas reveals two major limitations.

First, the “initial uptake of cleaner cooking systems” has been assumed to lead to sustained use and maintenance of cleaner cooking practices and subsequent health benefits that flow from sustaining cleaner cooking compared with traditional cook stoves [16]. The idea of “initial uptake” is merely the start of a process and is not adequate and indicative to conclude that cleaner cooking behavioral change has occurred, which in the long run delivers health, environmental, and climate benefits [7, 17, 18]. An appropriate approach is to emphasize “sustained adoption” of cleaner stoves and clean fuels rather than just the “initial uptake” of cleaner cooking technologies [6, 14].

Second, there is a lack of a systems perspective to understand sustained adoption of cleaner cooking systems. A systems perspective acknowledges that behavior is produced by interactions and feedback mechanisms among different sub-systems. The relationships between the different sub-systems or parts of a system are often non-linear and characterized by time delays. Perturbation in one part of the system can cause change in other parts as the cause and their effects are not proximate. A systematic review located 57 studies and listed 31 factors across 7 domains that potentially influence the adoption and sustained use of cleaner cooking technologies [19]. The authors of this review conclude that the magnitude of influence of a set of factors depends on the context of the situation [19]. The adoption and sustained use of cleaner cooking technologies by poor communities are functions of social, ecological, and technological interactions [19]. Explaining these factors and the interplay among them in the uptake and sustained use of cleaner cooking technologies and fuels needs a systems perspective. Systems thinking has been absent in examining the uptake, sustained use, and maintenance of cleaner stoves and cleaner fuels.

## Methods

### Study aim

This paper is part of a larger study aimed at understanding the underlying feedback mechanisms that cause the abandonment or sustained adoption of evidence-based cleaner cooking interventions (bio gas digesters and cleaner cookstoves) that reduce HAP and improve the respiratory health of women and children. This protocol paper will specifically highlight the process of involving communities at the center of the problem to elicit the underlying feedback mechanisms driving sustained use or disuse of cleaner cooking technologies.

### Study design

This study uses a comparative case study design to understand the dynamics of sustained use or abandonment of cleaner cooking technologies. In comparing these cases we are exploring the structure of feedback mechanisms that are likely to drive sustained adoption and abandonment of cleaner cooking systems overtime in these communities. With the use of multiple cases, protocols can be replicated and implemented for different types of cleaner cooking technologies to improve the generalizability of the results. A community that adopts a cleaner cooking technology and continues to use it is classified as a successful case; a community that adopts and then progressively abandons a cleaner cooking technology is an unsuccessful case.

Four communities in rural Rajasthan, India, were selected on the basis of the following criteria:

- Communities which are similar in socioeconomic status and geographically proximate to each other, so we are confident of similar livelihoods, ecosystems, and natural resources.
- Communities that are exposed to the same cooking technologies, and an installation of the cooking technologies were performed during the same year by the same local agency.

Based on our selection criteria, we included two communities located in Bhilwara district in which biogas disgesters were introduced at the same time. In these two communities, we examine biogas (methane anaerobic digester) adoption. One community sustainably adopted and used these biogas digesters with minimum stacking (successful case). While, the second community, only ten kilometers away in the same district, progressively abandoned these biogas digesters (unsuccessful case). In Udaipur district we selected two other communities that are similar in their socioeconomic and geographic characteristics, but differed in their adoption and sustained use of cleaner smokeless biomass cook stoves. In Udaipur, one community sustainably adopted these cleaner smokeless biomass cookstoves (successful case). While, the second community progressively abandoned these cookstoves (unsuccessful case). In all four communities cleaner cooking technologies were installed with help from our local partner agency, the Foundation for Ecological Security (FES), in 2004. Our model compares the two successful cases with the two unsuccessful cases. The criteria for comparison is the sustained adoption of cleaner cooking systems in two successful cases relative to progressive abandonment in the two unsuccessful communities. Our model reveals the underlying feedback mechanisms driving these communities to sustainably adopt (in the two successful cases) or progressively abandon (in the two unsuccessful cases) cleaner cooking systems. The comparative study design enables us to compare the communities both in terms of the behavior (i.e. sustained use and abandonment) and the underlying feedback mechanisms (i.e. structure). The goal is to compare the structures and understand how they produce two different behaviors: sustained use versus disuse over time.

### Overall approach

Our study uses a community based system dynamics (CBSD) modeling approach [20] to study barriers and enablers instrumental in sustained adoption or abandonment of cleaner cooking technologies in these four communities of rural Rajasthan, India. CBSD is a particular approach to building a system dynamics model of a behavioral trend with communities. System dynamics is a computational modeling technique that is used to illustrate and understand complex systems [21]. In particular, modeling system dynamics helps to understand the relationship between the underlying structure of feedback mechanisms and the system behavior produced by those mechanisms.

Traditionally, expert modelers in consultation with a few key stakeholders have built system dynamics models [22]. Realization of value addition in including stakeholders in the modeling process gave rise to Group Model Building (GMB) [22]. Building models using GMB involves carefully designed sessions with various stakeholders that come together and contribute to the model building process facilitated by a modeler. In short the group works to define the problem, specify the underlying feedback mechanism causing the problem, discuss ways to address the problem, and later the modelers test scenarios using the model [22]. Participation of stakeholders in the process of eliciting feedback mechanisms contributes to clarifying the problem and creating buy-in for the different recommendations. Those involved also gain significant insights regarding the system, which is otherwise mostly accessible to expert modelers [22].

GMB has mainly been developed through practice with key decision makers in organizations both government and private. When examining social problems in marginalized communities, it is essential to involve community members and not only organizations and professional working with communities on the problems [20, 22]. CBSD is therefore a particular approach to participatory modeling that provides community members who are embedded in the problem a voice in the modeling process [20]. Not only does this provide an opportunity to conceptualize the problem from the perspective of those involved in the problem but increases the face validity of the concepts and processes in the model [22]. A combination of systems perspective and community based approach allows us to look more closely at the relevant social, ecological, and technological systems driving a problem. The focus of CBSD is on understanding and solving problems that involve dynamic complexity [20].

In CBSD we elicit perspectives of key actors about the central problem that is complex and which could involve many interacting elements. Women are central to the problem of cleaner cooking and adoption of clean fuels in rural India as they are primarily responsible for cooking. Therefore, they are also responsible for the use and maintenance of cleaner cooking technologies. Our group model building sessions were primarily conducted with women. FES staff members who had working relationships with these communities recruited ten women from each village. The modeling sessions were held in a location away from the village at a community center to avoid possible distractions such as childcare and daily chores. Another possibility in the village was for men to join the meetings and the social norms would not have allowed us to exclude them. In the presence of men, rural women in Rajasthan defer to them and hesitate to share their opinions freely. A community center, away from the village provided a free and safe space for women to voice their opinions and engage in group model building. Although men rarely engage in cooking or the maintenance and upkeep of stoves, their opinions matter, especially because of their control over household decision making and resources. To understand the perspectives of all members of the community, we engaged both men and women from each of the four communities in the model validation stage.

### Research team roles

Model building is a team activity and a clear delineation of roles of members in the research team is crucial for successful CBSD modeling [20]. The following specific roles and descriptions are defined for each member of the research team (depending on field circumstances, one individual may fill multiple roles):

#### Community facilitator

A community facilitator acts as a bridge between the research team and the community. In this study, FES staff members who have been working in these communities for approximately a decade are the community facilitators. It is important for community facilitators to have prior relationship with community members; otherwise the level of engagement can be low, and narratives are shallow. FES team introduces the research team and describes the project to the community. They engage with the community members to help the research team better understand the problems and some of the underlying causes of the problems.

#### Modeler

A modeler listens to the conversation and the narrative and develops a representation of it in a system dynamics model. The modeler does not engage in community discussions. However, the community facilitators provide the modeler an opportunity to ask clarifying questions. In this study, research team members from Washington University are the modelers.

#### Note taker

The note-taker’s responsibility is to take detailed notes about what occurs in the community meetings. These notes are later used to provide a context for the system dynamics model.

#### Production coordinator

A model that is drawn in real time during a community meeting is then converted to a larger sheet of paper to share with the community for their feedback. The production coordinator makes sure that the model is comprehensible. In this study, a staff member from FES is the production coordinator as the models are translated and represented in Hindi with further explanation in the local dialect to the community.

#### Photographer

The photographer’s role is to visually record the group process, the community, and the cooking technologies. Even though photography is confined to general capture of community discussions and biogas digesters, it could be intrusive. In assigning a role to one person we ensure that photography is done in a non-interventionist way and from a distance. At the beginning of the group process, the community facilitators seek the consent of the group to take pictures.

#### Translator

When working in rural communities in India, there are multiple language barriers. Many understand Hindi but reply in their local dialect. Therefore, a translator in CBSD sessions in rural India must understand both the dominant regional language (Hindi, Telugu, or Oriya for example) and the spoken dialect of that particular community. Such a person translates for the modeler the discussion between the community members and the community facilitator.

### Action plan for conducting CBSD

**Fig. 1:**
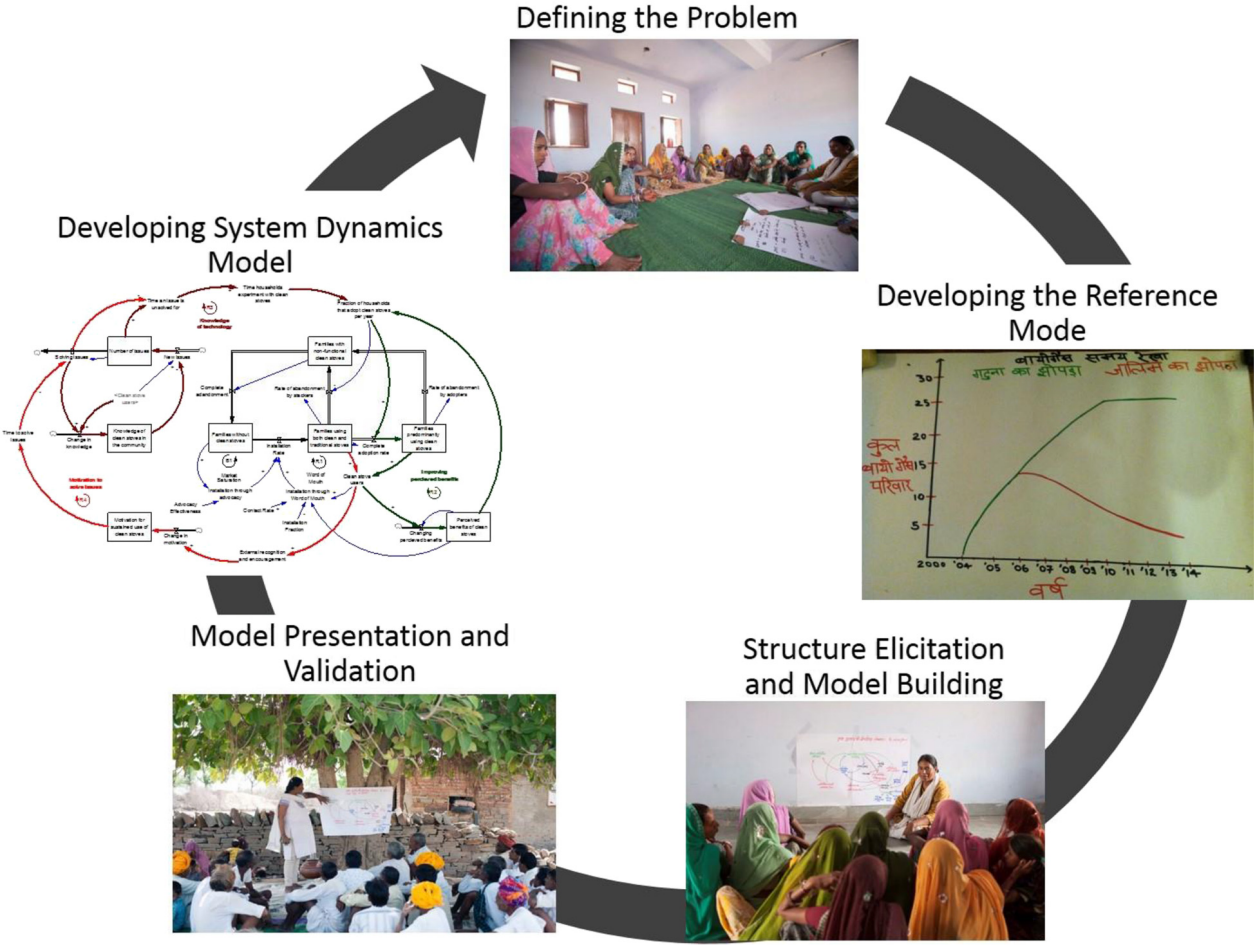
Sequence of stages in community based system dynamics and causal loop development

The following action plan provides a description of steps undertaken for conducting a CBSD. A diagrammatic sketch of the action plan for conducting CBSD is presented in Fig. 1. The figure shows the cyclical nature of the CBSD process. The insights from the model and the model building process are always aimed at an improved understanding of the problem. These steps are organized in a chronological manner and were repeated in all four communities included in the study.

#### Introduction

The community facilitator introduces the research team and the project. It is important to convey the message that the experience and knowledge of the community members are valuable to the researchers and that the researchers are in the community to learn and to help. A local field staff person plays the role of a community facilitator. That person has multiple years of experience working with the community and knows the community. The community facilitator begins the conversation by asking general questions about the community and cleaner cooking technologies. The need for the study is also explained to the community members along with an introduction of members of the research team.

#### Reference mode elicitation

In CBSD, reference modes are the temporal patterns of dynamic behavior produced by underlying feedback structures [20]. Reference modes define the problem, which the model will characterize and present. The reference mode in this project is the sustained adoption or progressive abandonment of cleaner cooking systems over time after their installation in 2004. In this study, our reference mode provides crucial information about the dynamic behaviors related to the adoption, use, and abandonment of cleaner cooking technologies. The goal of drawing a reference mode with the community members is to get a representation of the pattern of behavior over time but also to converge on a specific problem over time. Asking participants how many clean stove technologies were installed in a particular time helps in understanding the reasons for installing such stoves, how the initial idea of taking up a clean fuel or a stove came about. When participants provide information that point to initial uptake and then a declining trend in the community, the facilitator has an opening to ask why the numbers are in decline over time. People in the study communities are not familiar with calendar years (i.e. 2004, 2005, and so on). We used various frames of reference such as the ages of their children, critical events in their communities to facilitate our discussion along a time horizon. The familiarity of the facilitator with the community is helpful in interpreting a locally produced time horizon into years.

#### Structure elicitation

The community facilitator then explains that we want to understand from the community how different processes and events are consequential to the behavior shown in the reference mode. The discussion begins with simpler questions, such as why the community members adopted a cooking technology, the reasons for using them over time, or for abandoning them. The facilitator then goes deeper through probing. For example, if some community members say that they continue to use the technology because it is helpful and solves the household smoke problem, the community facilitator would then follow up by asking questions about how the community member learned to maintain the technology since it is so useful. The facilitator might further probe whether he or she is receiving external or in-community assistance with maintaining an anaerobic digester. The modelers and other team members do not contribute towards the interaction. If the facilitator is not sure how to move forward or the other team members wish to ask a question, a process is designed during the planning on how to deal with such situations. In this study, community facilitators were asked to direct the attention to other team members if there was an issue or towards the end of a set of questions when there is a natural break. For example, the community facilitator would say, “to ensure that we have covered all the points, let me turn to other members of the team if they have any questions or comments.”

#### Model building

During the structure elicitation phase, the modeler derives variables and causal mechanisms. The goal is to visually represent the narratives of the community members as causal mechanisms with feedback loops, referred to as a causal loop diagram (CLD). Model building and structure elicitation is an iterative process. As the modeler draws a CLD based on the narrative, the modeler may have questions or clarifications prompted by the emerging diagram. When the facilitator turns to team members and asks if they have any questions, the modeler is then free to ask clarifying or probing questions that lead to a CLD that is deeper or clearer. It is crucial to design some time during the process, where the members of the research team can come together to assess the progress [20]. This enables the team to evaluate the situation and think of action steps for the rest of the session. In this study, we conducted a morning session with a community lunch break. During the break, team members discussed the model and assessed if we needed more information. Once the community session reconvened, the community facilitator asked multiple questions based on team discussion during lunch break.

#### Model presentation and validation

Once structure elicitation is completed, the CLD is copied onto a larger sheet of paper and translated into the local language. When possible, symbols and icons were used in the CLD to convey the causal story. For example, alongside the variable “perceived benefits” a smiley face was drawn to represent satisfaction and good outcome. Presenting the model back to the community members is an important step in CBSD. Showing the model brings clarity to the community about what the modelers were doing during the community conversation. Presenting the model while the discussion is fresh in the community’s mind provides an ideal opportunity to make further changes. Model presentation begins with a discussion of the reference mode representing a problem over time. The pattern of behavior over time is explained and this also re-iterates that the goal is to understand the reasons for this behavioral trend. The community facilitator explains what the modeler just did and begins to re-narrate the stories with the use of the model. Community participants are encouraged to correct or add to the model at any point during the narration of the model. Their feedback can range from changing the causal links between variables, to changing the names of the variables, and adding or deleting variables.

#### Closing remarks

The community facilitator ends the session by thanking community participants and explaining the next steps of the project. Providing incentives to participate in such meetings is a complicated question and should be done with understanding of the local context. In discussions with FES we decided against providing monetary incentives for community participation. FES conducts many such meetings with the community in the region during the routine course of their work. Providing monetary incentives could create monetary expectations during future meetings. Instead we provided steel lunch boxes to all the participants to carry their lunch when they go to their fields or for daily wage labor.

### From causal loop diagram (CLD) to a system dynamics model

The participatory model building phase of the CBSD with the communities yields a reference mode and a causal loop diagram. The reference mode depicts the trends in sustained use and abandonment of clean cooking technology in the two communities. The CLD illustrates the feedback mechanisms posited to be the source of the two trends (sustained adoption or progressive abandonment of cleaner cooking systems). With multiple feedback processes it is impossible to infer the behavior based solely on a qualitative CLD [23]. Therefore, a system dynamics simulation model will be developed, in the next phase of this study, to test whether the feedback mechanisms in the CLD are capable of producing the two trends of sustained adoption and progressive abandonment overtime.

Developing a system dynamics model entails defining the feedback mechanisms as a set of non-linear differential equations. These differential equations are solved comuputationally to produce simulation results using Vensim DSS (Ventana Systems, UK) software. An array of tests are conducted to ensure the robustness of the model [24]. The model is then analyzed to understand how each of the feedback mechanisms contribute to the simulated behavior. This model development phase is now underway and the details of the process and results will be published in subsequent papers. The goal for the simulation model will be to replicate the reference mode representing both the sustained use and the abandonment of the cleaner cooking technologies, as we vary the underlying motivations driving the sustained use or abandonment of the stoves. The results from these simulations will facilitate analyses as well as the identification of potential interventions. The simulation and analyses of the simulation is performed by varying the parameters of the model and assessing the resulting effects on the reference mode.

## Limitations

This study explores the dynamics of sustained adoption and abandonment of cleaner cooking technologies and fuels in rural communities of India through a novel approach involving CBSD. However, there are some limitations that should be noted here.

First, there could be issues of recall bias in participating communities in the study thereby reducing the accuracy of their responses. However, the presence of many community members in a group setting facilitates self-correction and triangulation and minimizes the risk of recall bias [25, 26].

Second, vocal and active members of a community may shape the model-building process, thereby resulting in a “bandwagon effect” [20, 22]. Passive and reticent community members tend to support the views of their more active counterparts, which may reduce the validity of the final CLD. Community facilitators in the study were reminded to encourage reticent community members to participate in the model-building process. However, care should be taken to ensure that this occurs with minimal involvement of the community facilitators in a non-interventionist fashion [22].

Third, some variables in the final CLD may be subjective and based on community perceptions. The quantification of these variables in the SFD and the provision of parameters, initial values, and final values for such variables can be challenging. This is minimized by performing an extensive literature review and in having a deeper knowledge of the variables being studied within the context of the system being considered.

## Trial status

CBSD modeling is conducted with communities, and not with individual households or participants. The Institutional Review Board (IRB) of Washington University in St. Louis has exempted this type of modeling from review. Recruitment of communities and community modeling for this study began in May 2014 and concluded in June 2014. Model simulation and analyses are currently underway.

## Discussion

Social and behavioral factors cannot be ignored in the design and implementation of clean cooking systems to address HAP and associated health problems in poor communities [13]. In understanding the feedback between social, behavioral and other drivers of increased access to and use of clean cooking systems we increase the likelihood of identifying high leverage points of intervention to increase uptake and exclusive use of clean cooking. For effective translation of efficacious clean cooking technologies, the focus should shift to dissemination and implementation strategies of adapting and sustainably lodging them in the daily lives of the poor. This study protocol utilizes a novel approach of CBSD to reveal the underlying mechanisms driving sustained use or abandonment of clean cooking systems. A significant outcome of this novel approach will be the development of a simulation model of feedback mechanisms derived from using the protocol described in this paper. The model will be tested on its ability to reproduce system behavior and its logical consistency. Finally, the model will be used to conduct experiments regarding the impact of various interventions on sustained use of clean cooking technology. In underscoring the feedback mechanisms of enablers and barriers of sustained use of clean cooking systems, we advance our efforts to develop, tailor, and scale evidence-based dissemination and implementation strategies for the uptake and sustainment of clean cooking by rural poor in India.

## Abbreviations

CBSD: community based system dynamics
CLD: causal loop diagram
FES: foundation for ecological security
HAP: household air pollution
SFD: stock and flow diagram
